# Opportunistic infections in patients treated with immunotherapy for cancer

**DOI:** 10.1186/2051-1426-2-19

**Published:** 2014-06-18

**Authors:** Chrisann Kyi, Matthew D Hellmann, Jedd D Wolchok, Paul B Chapman, Michael A Postow

**Affiliations:** 1 New York-Presbyterian Hospital, Weill Cornell Medical College, 525 East 68th Street, New York, NY 10065, USA; 2 Memorial Sloan-Kettering Cancer Center, Melanoma and Immunotherapeutics Oncology Service, 300 East 66th Street, New York, NY 10065, USA; 3 Memorial Sloan-Kettering Cancer Center, 1275 York Avenue, New York, NY 10065, USA

**Keywords:** Immunotherapy, Immune-related adverse events (irAEs), Opportunistic infections, Malignancy, Melanoma, Ipilimumab

## Abstract

Immunomodulatory antibodies that enhance the immune system to fight cancer are revolutionizing the treatment of patients with an expanding variety of malignancies. There is a unique spectrum of side effects associated with immunomodulatory antibodies, termed immune-related adverse events (irAEs), which include colitis and hepatitis among others. The treatment of refractory or severe irAEs can occasionally require significant immunosuppression, involving steroids or tumor necrosis factor-alpha antagonists, placing these patients at risk for infections. We present the first reported case to our knowledge of an opportunistic infection in a patient treated with an immunomodulatory antibody. As the use of immunomodulatory antibodies expands and more patients develop irAEs that require treatment with immunosuppression, recognition of the potential for opportunistic infections in this emerging patient population will be critical. Prospective trials are needed to define the optimal immunosuppressive management of irAEs and determine whether prophylactic antiviral, antibacterial, or antifungal therapies are beneficial in this unique population.

## Background

T-cell checkpoint blockade is one of the most active areas of cancer research and has demonstrated dramatic results in patients with a variety of cancers [1-5]. Ipilimumab, an antibody against cytotoxic T-lymphocyte-associated antigen 4, was the first immunomodulatory checkpoint inhibitor approved by the US Food and Drug Administration for patients with advanced melanoma [1]. Programmed cell death-1 (PD-1) receptor, another immunologic checkpoint, has also shown great promise as a therapeutic target in a variety of malignancies [4-6].

T-cell checkpoint blockade can result in side effects called immune-related adverse events (irAEs), most commonly involving the skin (rash) and, particularly with ipilimumab, gastrointestinal tract (diarrhea, hepatitis). Refractory or severe irAEs often require treatment with prolonged immunosuppression including high-dose steroids and sometimes the addition of tumor necrosis factor-alpha blockade or other immunosuppressants [7]. We report a case of a patient with metastatic melanoma who was treated with ipilimumab, developed immune-mediated colitis requiring treatment with steroids and infliximab, and later developed an opportunistic infection with Aspergillus. This is the first case to be reported to our knowledge that specifically highlights the potential for developing opportunistic infections in this emerging patient population.

## Case presentation

A 48-year-old Caucasian man was diagnosed with primary cutaneous melanoma in 1991 when he was noted to have a pigmented lesion on his posterior right neck. Subsequent biopsy showed Breslow depth 2 mm melanoma. No information about the presence or absence of ulceration was available. He underwent wide local excision with pathology demonstrating no residual disease. A sentinel lymph node biopsy was not pursued.

The patient remained free of disease for 20 years until the age of 68 when he developed new hypertension, prompting a renal ultrasound that showed normal kidneys. Two hepatic lesions were incidentally noted. A subsequent positron emission tomography (PET) scan demonstrated bilobar hepatic metastases and multiple pulmonary metastases concerning for metastatic disease. Core biopsy of a lesion in the right hepatic lobe confirmed metastatic melanoma. Mass-spectrometry genotyping (Sequenom) revealed no known mutations that affect the gene encoding serine-threonine protein kinase BRAF (e.g., the *BRAF* V600E mutation).

Three weeks later, the patient began treatment with ipilimumab (3 mg/kg). After three doses of ipilimumab (approximately two months of therapy), he developed significant diarrhea. Colonoscopy with biopsy showed active colitis. He received two doses of infliximab (5 mg/kg, separated by 9 days) and high-dose systemic corticosteroids (methylprednisolone 2 mg/kg/day for one day, followed by prednisone 1 mg/kg/day tapered over one month) with ultimate resolution of his diarrhea.

**Figure 1:**
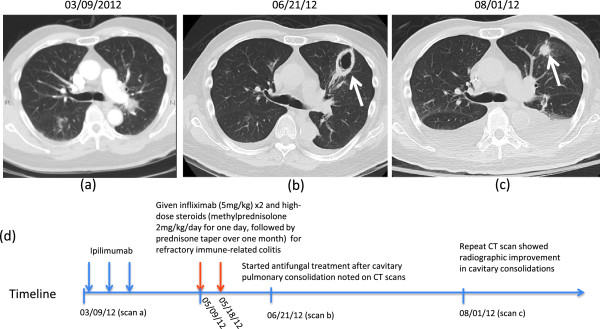
**Images and timeline of Aspergillus infection in patient treated with steroids for management of an immune-related adverse event. (a)** Baseline chest CT scan prior to ipilimumab. **(b)** Three weeks after receiving high-dose immunosuppression for immune-related colitis, CT scans showed cavitary pulmonary consolidations (white arrow). Subsequent bronchoalveolar lavage was consistent with *Aspergillus fumigatus* pneumonia. **(c)** After a 14-day treatment with antifungals, repeat CT scan showed radiographic improvement in cavitary consolidations, but increased bilateral pleural effusions. **(d)** Timeline of described events (not to scale).

A computerized tomography (CT) scan three months after starting ipilimumab demonstrated response of pulmonary and hepatic metastases. However, new bilateral cavitary pulmonary consolidations were noted concerning for fungal pneumonia (Figure 1a-b). At this time, the patient had no cough, fever, shortness of breath, or other pulmonary symptoms. Bronchoscopy was performed and bronchoalveolar lavage revealed *Aspergillus fumigatus* pneumonia with a lavage fluid also positive for galactomannan. Voriconazole and liposomal amphotericin B treatment for a 14-day course resulted in ultimate radiographic improvement (Figure 1c). Although his response to ipilimumab lasted approximately six months, he later had disease progression and unfortunately passed away due to metastatic disease.

## Conclusions

As the use of immunomodulatory antibodies that block T-cell checkpoints expands, so too may the complications associated with this treatment. The unique spectrum of immune-mediated toxicities from these agents has been well characterized and algorithms for suggested immunosuppression regimens have been developed. However, the potential for opportunistic infections to arise as a result of the immunosuppression necessary to treat an irAE has not previously been highlighted. Though we have chosen to describe this one illustrative case, we have observed additional cases at our institution, including patients with Fournier’s gangrene and cytomegalovirus viremia. Clinicians across the spectrum of internal medicine must have a high degree of suspicion for the development of these rare infections as early recognition, diagnosis, and treatment are essential to achieve favorable clinical outcomes.

Consensus guidelines instruct clinicians on the prophylaxis and treatment of opportunistic infections arising in patients following hematopoietic stem cell transplantation [8]. As we learn more from patients treated with these novel immunomodulatory antibodies, similar guidelines may be necessary to define the optimal management strategies for irAEs while also minimizing infectious complications in this unique patient population. Ultimately, prospective trials may be needed to optimize the management of irAEs, taking into account the associated secondary infectious risks.

## Consent

Written informed consent was obtained from the patient’s next of kin for publication of this case report and any accompanying images. A copy of the written consent is available for review by the Editor-in-Chief of this journal.

## Abbreviations

CTLA-4: Cytotoxic T-lymphocyte-associated antigen 4; PD-1: Programmed cell death-1; irAE: Immune-related adverse event.

## Competing interests

JDW and MP receive research support from Bristol-Myers Squibb and have served on advisory councils. CK, MDH, and PBC have no competing interests to disclose.

## Authors’ contributions

CK and MAP conceived of this study report, collected the data, wrote and revised the manuscript. MH conceived this study report concept and reviewed the manuscript. JDW and PBC reviewed the manuscript. All authors read and approved the final manuscript.
